# Probiotic and Postbiotic Potentials of *Enterococcus faecalis* EF-2001: A Safety Assessment

**DOI:** 10.3390/ph17101383

**Published:** 2024-10-17

**Authors:** Kwon Il Han, Hyun-Dong Shin, Yura Lee, Sunhwa Baek, Eunjung Moon, Youn Bum Park, Junhui Cho, Jin-Ho Lee, Tack-Joong Kim, Ranjith Kumar Manoharan

**Affiliations:** 1 Research and Development Center, Bereum Co., Ltd., Wonju 26361, Republic of Korea; kihan@bereum.com (K.I.H.); hshin@bereum.com (H.-D.S.); lyr@bereum.com (Y.L.); an20112246@bereum.com (S.B.); moonej@bereum.com (E.M.); pyb12345@bereum.com (Y.B.P.); jhcho@bereum.com (J.C.); 2 Division of Biological Science and Technology, Yonsei University, Wonju 26493, Republic of Korea; drlogos@naver.com

**Keywords:** Enterococcus, postbiotics, EF-2001, phenotypic, safety assessment

## Abstract

Background: Probiotics, which are live microorganisms that, when given in sufficient quantities, promote the host’s health, have drawn a lot of interest for their ability to enhance gut health. *Enterococcus faecalis*, a member of the human gut microbiota, has shown promise as a probiotic candidate due to its functional attributes. However, safety concerns associated with certain strains warrant comprehensive evaluation before therapeutic application. Materials and Methods: In this study, *E. faecalis* EF-2001, originally isolated from fecal samples of a healthy human infant, was subjected to a multi-faceted assessment for its safety and probiotic potential. In silico analysis, CAZyme, biosynthetic, and stress-responsive proteins were identified. Results: The genome lacked biogenic amine genes but contained some essential amino acid and vitamin synthetic genes, and carbohydrate-related enzymes essential for probiotic properties. The negligible difference of 0.03% between the 1^st^ and 25^th^ generations indicates that the genetic information of the *E. faecalis* EF-2001 genome remained stable. The live *E. faecalis* EF-2001 (*E. faecalis* EF-2001L) demonstrated low or no virulence potential, minimal D-Lactate production, and susceptibility to most antibiotics except some aminoglycosides. No bile salt deconjugation or biogenic amine production was observed in an in vitro assay. Hemolytic activity assessment showed a β-hemolytic pattern, indicating no red blood cell lysis. Furthermore, the EF-2001L did not produce gelatinase and tolerated simulated gastric and intestinal fluids in an in vitro study. Similarly, heat-killed *E. faecalis* EF-2001 (*E. faecalis* EF-2001HK) exhibits tolerance in both acid and base conditions in vitro. Further, no cytotoxicity of postbiotic EF-2001HK was observed in human colorectal adenocarcinoma HT-29 cells. Conclusions: These potential properties suggest that probiotic and postbiotic *E. faecalis* EF-2001 could be considered safe and retain metabolic activity suitable for human consumption.

## 1. Introduction

In recent years, the exploration of probiotics has grown rapidly, driven by a growing recognition of their potential to modulate gut microbiota and confer health benefits [1]. These live microorganisms, administered in sufficient quantities, offer a promising avenue for maintaining gut homeostasis, bolstering immune function, and mitigating gastrointestinal ailments [2]. Among the numerous microbial candidates under investigation, strains of the genus Enterococcus have emerged as particularly intriguing contenders due to their resilience within the gastrointestinal milieu and purported health-promoting attributes [3].

*Enterococcus faecalis*, a constituent of the human gut microbiota, has garnered substantial attention as a potential probiotic agent. Its capacity to endure the rigorous conditions of the gut environment, adhere to intestinal epithelial cells, and modulate host immune responses positions it as a compelling candidate for therapeutic intervention [4]. However, amid the attention surrounding probiotic exploration, concerns regarding safety show large, particularly considering the virulence traits, antibiotic resistance profiles, and potential biogenic amine production observed in certain strains of *E. faecalis* [5]. *E. faecalis* is known for some of its pathogenicity and multidrug resistance mechanisms in humans [6]. On the other hand, *E. faecalis* is naturally found in healthy humans and can be utilized as a probiotic. Notably, Symbioflor1 and *E. faecalis* EF-2001 are characterized as probiotic strains by the absence of genes linked to drug resistance and pathogenesis [7]. The potential of Enterococcus species, including *E. lactis* and *E. faecium*, as probiotic strains has garnered substantial attention following recent studies. Probiotics have historically been linked to genera such as Bifidobacterium and Lactobacillus. Nevertheless, current studies have demonstrated that Enterococcus species can have advantageous probiotic qualities despite their historical link with disease [8,9,10].

Besides benefits, there are limitations in using live probiotic strains, such as concerns about their viability and stability [11]. To overcome these limitations, researchers have turned their attention to nonviable components of microbiomes, leading to the emergence of a new term called postbiotics [12]. Postbiotics refer to non-viable microbial cells, components, or metabolic byproducts that benefit human health. These postbiotics may include heat-killed cells, cell extracts, cell wall components, and secreted metabolites. By focusing on postbiotics, researchers can harness the health-promoting properties of microorganisms without the need for live cells [13]. This approach has several advantages. First, postbiotics are inherently stable and can be easily stored and transported, making them more convenient for use in various applications [14]. Second, the use of postbiotics eliminates the risk of infection or adverse reactions that may be associated with live probiotic strains. Third, postbiotics offer a broader range of therapeutic possibilities than live probiotic strains [15]. Taken together, *E. faecalis* EF-2001HK products have been reported as having several advantages in vitro and in vivo studies [16,17]. For instance, *E. faecalis* EF-2001HK effectively helps in biological activities such as anti-inflammatory effect, irritable bowel syndrome, acute gastric ulcer, and controls lipid metabolisms in vivo model [16,18,19,20]. Interestingly, *E. faecalis* EF-2001, both live and heat-killed cells, attenuates benign prostatic hyperplasia in an animal model from prostate enlargement [21].

Probiotics have traditionally been found in dairy products and fermented foods like cheese, yogurt, and kimchi, which are rich in beneficial bacteria that have been used for centuries to promote health [22]. However, a new and exciting approach to gut health involves the isolation of probiotic strains, such as *E. faecalis* EF-2001L, from the healthy feces of infants [23]. The identification of *E. faecalis* EF-2001L from the healthy infant gut offers an alternative strategy for developing probiotics based on bacteria that naturally inhabit the human gastrointestinal tract [24,25]. In response to these concerns, a severe and comprehensive safety assessment becomes imperative, serving as a critical precursor to the therapeutic application of *E. faecalis* strains [26]. Such assessments necessitate a multidisciplinary approach, integrating genomic analyses, in vitro assays, and microbial interaction studies to elucidate the safety profile and probiotic potential of candidate strains [27]. By particularly scrutinizing these attributes, researchers can delineate the therapeutic potential of *E. faecalis* strains, thereby guiding the development of probiotic formulations for human health applications.

In this study, we set out a detailed safety assessment of EF-2001L, isolated from the fecal samples of a healthy human infant [7]. Leveraging a multidisciplinary approach encompassing genomic analyses, in vitro assays, and microbial interaction studies, we try to evaluate EF-2001L comprehensively. Our endeavors are driven by the overarching goal of elucidating *E. faecalis* EF-2001′s safety profile and probiotic potential, thereby contributing to the broader understanding of *E. faecalis* EF-2001 as a probiotic agent. Through comprehensive examination evaluation, we aim to provide a strong foundation for the further exploration of *E. faecalis* EF-2001 in clinical settings, fostering its potential translation into therapeutic applications for human health.

## 2. Results

### 2.1. Genetic Stability Test

The genetic stability of *E. faecalis* EF-2001L was assessed by performing whole genome sequencing (WGS) on the 1^st^ and 25^th^ generations of the strain. A combination of short-read and long-read sequencing techniques was used to obtain comprehensive genomic data, followed by bioinformatics and comparative genomics analyses (Table 1). The results showed a negligible difference of 0.03% between the two generations, which can be attributed to sequencing errors or minor evolutionary changes. This minimal variation indicates that the genetic information of *E. faecalis* EF-2001L remained stable throughout 25 generations of cultivation. Additionally, a phylogenetic tree was constructed using three *E. faecalis* EF-2001L (original and 1^st^ and 25^th^ generation) sequences, further supporting the strain’s genetic stability (Figure S1).

### 2.2. Carbohydrate Metabolism Activity of E. faecalis EF-2001L

To investigate the carbohydrate metabolism activity of *E. faecalis* EF-2001L, its genome was analyzed using the CAZy database, which identified 80 CAZy families (Table 2). Specifically, the genome includes 2 auxiliary activities (AAs), 5 carbohydrate-binding modules (CBMs), 5 carbohydrate esterases (CEs), 49 glycoside hydrolases (GHs), and 19 glycosyltransferases (GTs). However, no polysaccharide lyases (PLs) were detected in the *E. faecalis* EF-2001L genome (Table 2). The presence of these diverse CAZy families suggests that *E. faecalis* EF-2001L has a strong capacity for carbohydrate metabolism, which is essential for its energy production and growth. This metabolic versatility could be advantageous for various applications, including bioprocessing, fermentation, and the development of functional probiotic products.

### 2.3. Biosynthetic Genes in E. faecalis EF-2001L Genome

The genome of *E. faecalis* EF-2001L contains numerous biosynthetic genes, including those involved in the synthesis of essential amino acids and vitamins, as detailed in Table 3. Specifically, genes associated with the production of essential amino acids were identified, such as those encoding tryptophan synthase, methionine transporter, arginine decarboxylase, and threonine synthase. Similarly, genes related to the biosynthesis of vitamins, including thiamine, biotin, and folate, were also detected. The presence of these biosynthetic pathways underscores the potential of *E. faecalis* EF-2001L as a probiotic strain, capable of contributing to essential nutrient synthesis.

### 2.4. Stress-Responsive Genes in E. faecalis EF-2001L

Stress response is a crucial characteristic of the probiotic strains, helping them survive and function effectively in various challenging environments. In this study, we identified key stress-responsive genes and their associated proteins within the *E. faecalis* EF-2001L genome. These proteins play significant roles in managing oxidative, heat, and osmotic stress.

Table 4 lists the proteins directly associated with these stress response genes. For instance, we identified glutathione synthetase (gshB) as a key player in combating oxidative stress [28]. Similarly, the heat shock protein GrpE is crucial for managing heat stress [29], while Aquaporin Z plays a vital role in responding to osmotic stress [30]. These findings highlight the strong stress response mechanisms in *E. faecalis* EF-2001L, emphasizing its potential effectiveness as a probiotic.

### 2.5. Phenotypic Safety Assessment

**Figure 1:**
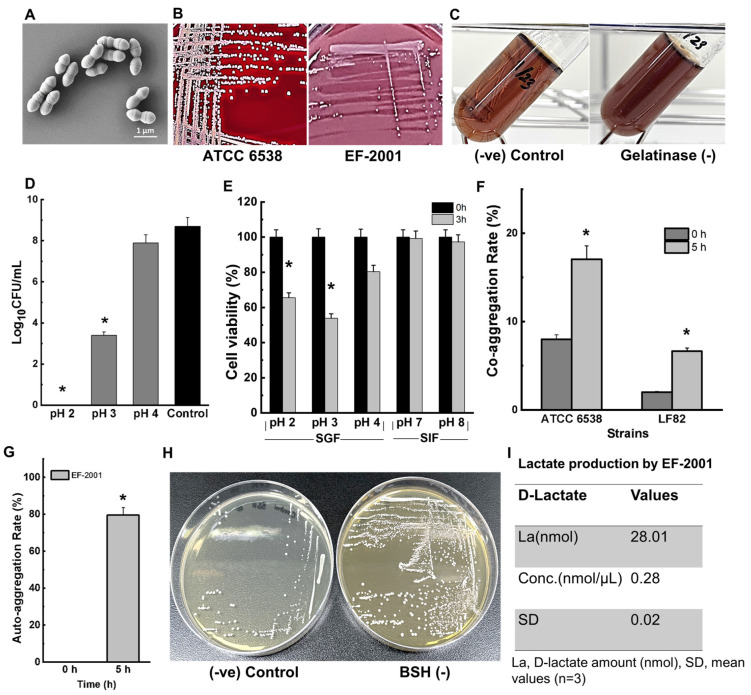
Phenotypic safety assessment of EF-2001L. (**A**) Cell morphology by SEM analysis. (**B**) Hemolytic activity of *E. faecalis* EF-2001L and *S. aureus* ATCC 6538 (positive control). (**C**) Gelatinase activity of *E. faecalis* EF-2001L. (**D**) Acid tolerance. Control refers to neutral pH. (**E**) Survival of *E. faecalis* EF-2001L in simulated gastric fluid (SGF) and simulated intestinal fluid (SIF). (**F**,**G**) Co-aggregation and auto-aggregation abilities. * *p* < 0.05 a significant difference between corresponding values at 0 h and treatment time intervals. Each experiment was performed using three independent cultures. (**H**). Bile salt deconjugation test. (**I**) D-lactate production assay. −ve and +ve denote negative and positive, respectively.

*E. faecalis* EF-2001L was evaluated for its antibiotic susceptibility using 10 different antibiotics (Table 5). The strain was found to be susceptible to most antibiotics according to established cutoff values. However, *E. faecalis* EF-2001L exhibited resistance to Kanamycin and Streptomycin. Scanning electron microscopy (SEM) images revealed that *E. faecalis* EF-2001L are ovoid and arranged in pairs or chains (Figure 1A). The strain showed no hemolytic activity, as evidenced by the lack of clear zones around colonies on blood agar plates (Figure 1B), and no gelatinase activity (Figure 1C). In contrast, the positive control, *S. aureus* ATCC 6538, exhibited β-hemolysis (Figure 1B).

### 2.6. Survival in Simulated Human Intestinal Environment

To assess the survival of *E. faecalis* EF-2001L in conditions mimicking the human intestinal environment, we conducted experiments across a pH range from 2 to 8. Initial testing under acidic conditions (pH 2, pH 3, and pH 4) revealed that *E. faecalis* EF-2001L cells fully survived at pH 4, while no cells survived at pH 2 (Figure 1D). A significant reduction in cell viability was observed at pH 3 after 24 h (Figure 1D).

Further experiments simulated the intestinal environment using artificial gastric fluid (SGF) and simulated intestinal fluid (SIF) at pH levels 2, 3, 4, 7, and 8. Notably, *E. faecalis* EF-2001L cells survived in SGF at pH 2 after 3 h (Figure 1E). However, cell viability was significantly inhibited under these SGF conditions, whereas the cells completely survived in SIF, indicating a healthy survival mechanism in the simulated intestinal environment (Figure 1E).

### 2.7. Auto- and Co-Aggregation

To evaluate the intestinal adhesion of probiotic strain, *E. faecalis* EF-2001L was used to examine the auto-aggregation and co-aggregation with two pathogenic strains, *S. aureus* ATCC 6538 and *E. coli* LF82. It was noted that co-aggregation with *S. aureus* ATCC 6538 and *E. coli* LF82 was 17% and 6.6%, respectively (Figure 1F). Whereas auto-aggregation of *E. faecalis* EF-2001L was 80% after 5 h (Figure 1G).

### 2.8. Bile Salt Deconjugation

*E. faecalis* EF-2001L was tested for its ability to deconjugate bile salts using 0.5% sodium taurodeoxycholate. The results indicated that *E. faecalis* EF-2001L did not exhibit bile salt deconjugation activity, as no clearing zones were observed around the *E. faecalis* EF-2001L colonies on MRS agar plates containing 0.5% taurodeoxycholic acid (Figure 1H). This suggests that *E. faecalis* EF-2001 is unable to deconjugate bile salts under the tested conditions, confirming its safety and potential as a probiotic strain.

### 2.9. D-Lactate Formation

To evaluate D-lactate production by *E. faecalis* EF-2001L, a D-lactate Assay Kit (colorimetric) was utilized. This analysis is particularly relevant for individuals with conditions like short bowel syndrome or certain gastrointestinal disorders, who may be sensitive to D-lactate accumulation. The results showed that *E. faecalis* EF-2001L produced very low levels of D-lactate, averaging 0.28 nmol/µL (Figure 1I). These findings indicate that *E. faecalis* EF-2001L produces minimal and non-harmful levels of D-lactate under the specified conditions, supporting its suitability as a safe probiotic.

### 2.10. Carbohydrate Utilization of E. faecalis EF-2001L

**Figure 2:**
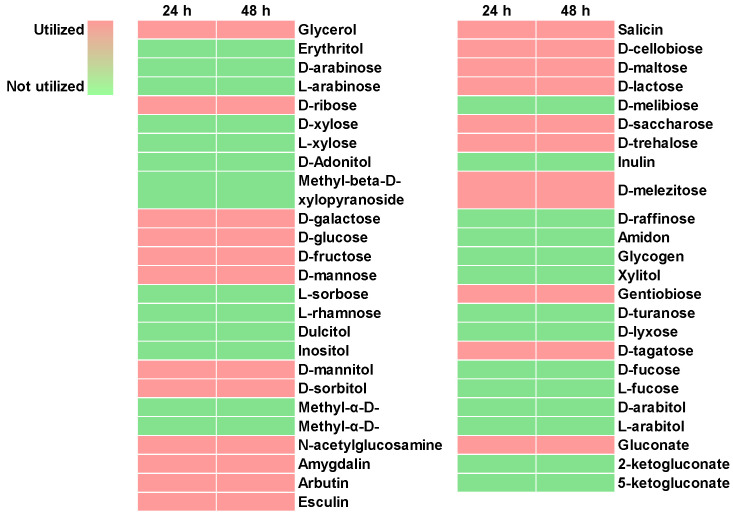
Carbohydrate utilization of *E. faecalis* EF-2001L.

To evaluate the carbohydrate fermentation capabilities of *E. faecalis* EF-2001L, an API 50 CHL/CHB kit (BioMérieux) containing 49 selected carbohydrate sources was used. EF-2001L demonstrated the ability to utilize several C6 sugars, including D-glucose, D-galactose, D-fructose, and D-mannose. Additionally, it could ferment sugar alcohols such as glycerol, D-mannitol, and D-sorbitol. The strain was also capable of metabolizing disaccharides like D-lactose, D-sucrose, D-trehalose, D-melezitose, D-gentibiose, and D-tagatose. However, *E. faecalis* EF-2001L did not metabolize D-ribose, a C5 sugar (Figure 2).

The ability of *E. faecalis* EF-2001L to utilize a wide range of carbohydrates suggests that it can efficiently convert these sources into energy, which is essential for its growth and the potential production of beneficial bioactive compounds. This capability indicates that *E. faecalis* EF-2001L could be highly valuable in applications such as bioprocessing, fermentation, and the development of probiotic products.

### 2.11. Biogenic Amine Production

Amino acid decarboxylases are expressed by several groups of microorganisms, including Enterobacteriaceae, Micrococcaceae, *Pseudomonas* spp., *Bacillus* spp., and many lactic acid bacteria (LAB). The ability of *E. faecalis* EF-2001L to produce biogenic amine was tested qualitatively by decarboxylase plates containing six amino acids. None of these amines detected were produced by *E. faecalis* EF-2001L (Table S1).

### 2.12. Hyaluronidase Activity

Hyaluronidase, a degrading mucopeptide called hyaluronic acid (HA), is produced by pathogenic strains such as streptococci, staphylococci, pneumococci, and Clostridia [31]. *Enterococcus* Spp. was reported from several studies to hold genes encoding virulence factors in addition to the hyaluronidase [32]. In this study, *E. faecalis* EF-2001L was tested to determine the production of hyaluronidase by observing a clearing zone around an inoculated area on an HA mixed BHI agar plate. As a result of the experiment, the clearing zone around the inoculated *E. faecalis* EF-2001L was not observed, while *S. aureus* ATCC 6538 showed the clearing zone around the colony. This result indicates that *E. faecalis* EF-2001L does not produce hyaluronidase (Table S2).

### 2.13. In Vitro Safety Assessment for E. faecalis EF-2001HK

**Figure 3:**
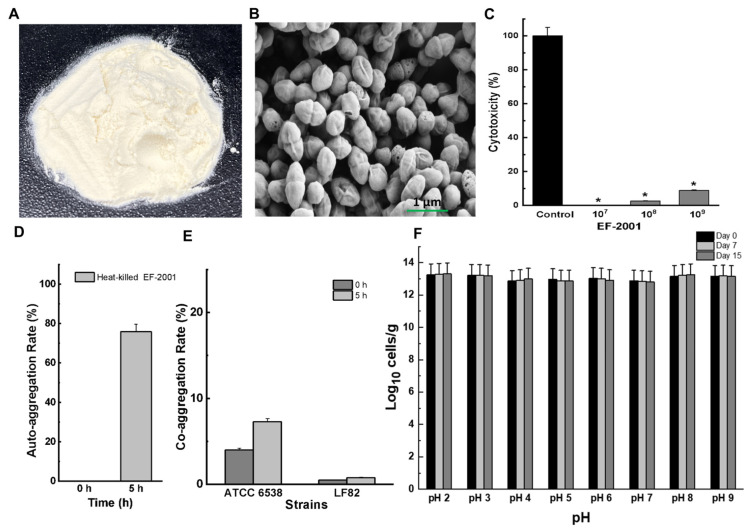
Safety assessment of EF-2001HK *(***A**) Heat-killed probiotic powder (*E. faecalis* EF-2001 HK) (**B**) Cell morphology by SEM analysis. (**C**) Cytotoxicity of *E. faecalis* EF-2001HK using human colorectal adenocarcinoma HT-29 cells. * *p* < 0.05 vs untreated control. (**D**,**E**) Auto-aggregation and co-aggregation abilities. (**E**,**F**) Log10 value of *E. faecalis* EF-2001HK cell numbers per gram exposed in pH 2 to 9 for respective days.

The postbiotic heat-killed *E. faecalis* EF-2001 (EF-2001HK, postbiotic product from Bereum Co. Ltd, Wonju, Republic of Korea) has been evaluated for in vitro safety assessment (Figure 3A). SEM was used to observe morphological changes in *E. faecalis* EF-2001L cells after heat treatment. The cells appeared shrunken, and their surface morphology showed no significant alterations due to heat shock (Figure 3B).

To develop heat-killed or postbiotics as safe and effective products for human consumption, we conducted further safety assessments, including cytotoxicity, auto-aggregation, co-aggregation, and acid tolerance tests. Figure 3C demonstrates that *E. faecalis* EF-2001HK exhibited almost no toxicity against human HT-29 cells, even at concentrations of 10^7^ and 10^8^ cells. In particular, the treatment of HT-29 cells with 10^7^, 10^8^, and 10^9^ cells/well of *E. faecalis* EF-2001HK resulted in a 0.0%, 2.6%, and 8.8% increase in the amount of LDH in the medium, respectively, indicating no significant cytotoxicity. Additionally, auto-aggregation of *E. faecalis* EF-2001HK reached 75% after 5 h (Figure 3D), while co-aggregation with *S. aureus* ATCC 6538 and *E. coli* LF82 was 7% and 1%, respectively (Figure 3E). Interestingly, there was no significant difference in cell numbers when exposed to various pH conditions ranging from pH 2 to pH 9 (Figure 3F).

## 3. Discussion

In the current observation of probiotic research, the safety and efficacy of specific strains are of particular interest, especially given the recognized health-promoting potential of probiotics when administered in appropriate quantities [33]. In this context, the *E. faecalis* EF-2001L, isolated from the feces of a healthy infant, emerges as a highly relevant candidate for further probiotic research. Enterococcus species, particularly *E. faecalis* and, to a lesser extent, *E. faecium*, are among the initial microorganisms to colonize the digestive systems of most healthy, breastfed infants within the first 7 to 10 days after birth [34,35]. This early colonization, largely derived from maternal flora, highlights the critical and beneficial role these bacteria play in establishing gut health [36]. Given its origin and the inherent functional attributes of *E. faecalis*, the EF-2001L strain aligns closely with the key characteristics sought in probiotics, particularly in supporting and enhancing gut health. This makes it a strong candidate for further investigation in the development of effective probiotic therapies [35].

Genomic analysis of *E. faecalis* EF-2001L has provided key insights, revealing the presence of genes coding for biosynthetic pathways and enzyme systems that could augment gut integrity and function [37]. L-arginine, L-histidine, L-lysine, L-ornithine, L-tryptophan, and L-tyrosine are main sources of biological amines in foods [38]. Most importantly, genes involved in the production of biogenic amines are absent from the strain’s genome. This lack of evidence suggests a lower chance of adverse effects and supports the strain’s safety for use in the future by reducing concern over potential health risks related to amine synthesis [39]. Previous research has also demonstrated similar findings, indicating that *Enterococcus lactis* is suitable for food processing due to its inability to produce biogenic amines [10]. In our further evaluation of mobile genetic elements (MGEs) in the *E. faecalis* EF-2001L genome, we discovered 17 MGEs (Table S3). No resistance profiles were linked to these MGEs within the *E. faecalis* EF-2001L chromosome, as reported by Mikalsen et al. [40]. To understand the risk of horizontal gene transfer, we identified the MGEs and mapped their positions in the genome. Interestingly, none of these MGEs were located near the resistance gene *lsa(A)*. The positions of the resistance gene and the nearest MGEs are shown in Figure S2. Since the gene loci and MGE loci are not close to each other, the risk of horizontal gene transfer is minimal [41].

*E. faecalis* EF-2001L also presents a convincing safety profile based on its minimal virulence potential, limited production of D-lactate, and general antibiotic susceptibility though resistance to few aminoglycosides [42]. Observations of no bile salt deconjugation and an absence of hemolytic activity not only reinforce its safety but also suggest a lower likelihood of interference with the host’s metabolic pathways, a notable consideration given the adverse outcomes from bile salt deconjugation and hemolysis observed in other contexts.

Examinations of *E. faecalis* EF-2001L’s responses to in vitro conditions simulating the human gastrointestinal environment additionally confirmed its viability, adding to the strain’s credentials as a beneficial microbial supplement [16]. The findings of this study are consistent with earlier studies that showed the high gastrointestinal survivability of *E. faecalis*, indicating the possibility of using certain strains as probiotics [22]. Even though *E. faecalis* EF-2001L displayed minimal activity against oral candida strains [43], which could be viewed as less than optimal for a probiotic seeking to exert antimicrobial effects, such restraint could suggest a nuanced interaction with the host’s indigenous microbes and an emphasis on maintaining microbiome balance [44].

**Figure 4:**
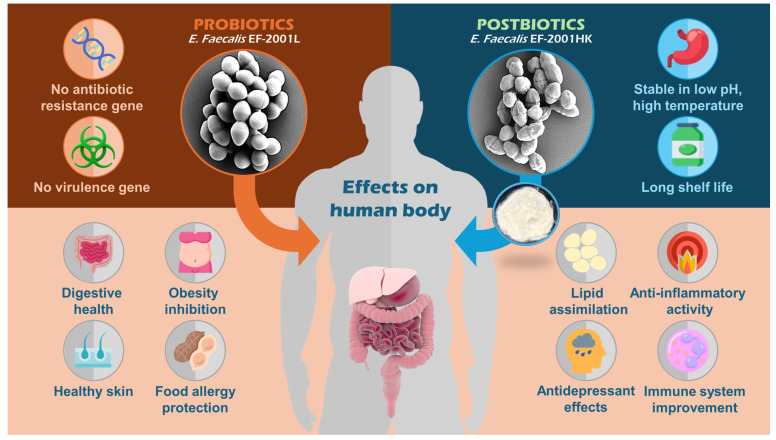
Schematic diagram of probiotic and postbiotic properties of *E. faecalis* EF-2001.

The specific assessment of the heat-killing procedure’s efficiency, which effectively inactivated probiotic cells within a brief exposure time, suggests that *E. faecalis* EF-2001HK cells can be considered for use where living bacteria might pose risks or are otherwise contra-specified [45]. However, while this indicates considerable safety, it also raises questions about the functional capabilities of dead cells within the human gastrointestinal system. There are limited studies on the effects of heat-killed probiotic strains, but some have shown promising results in supporting immune function and balancing the human intestinal microbiota [46,47]. These studies also suggest that heat-killed probiotics may enhance immune responses and improve stress tolerance. However, there are challenges with using inactivated probiotics, such as their inability to adhere to the intestinal tract effectively, and the increased cost of long-term use. These issues have led some researchers to propose postbiotics as a more viable alternative for supporting gut health, as they may offer similar benefits without the same limitations. Therefore, we have assessed both live and heat-killed cells through in vitro and in vivo studies to address the limitations associated with using Enterococcus as a probiotic. Considering the safety profile of *E. faecalis* EF-2001L in this study, the commercialized product from *E. faecalis* EF-2001HK shows promise for human applications. Given its diverse effects on improving gut health and enhancing biological activities in animal models [21,48], it is essential to validate *E. faecalis* EF-2001HK for commercial use. In Figure 4, we have illustrated the potential health impacts of *E. faecalis* EF-2001, both as probiotic and postbiotic, in a schematic diagram. The figure highlights that *E. faecalis* EF-2001 can be consumed in both forms and demonstrates that it exhibits similar biological activities in vitro and in vivo. The EFSA guidance on assessing the safety of live microbes is quite challenging and lacks clear, straightforward guidelines. Recent studies suggest that ensuring the safety of potential postbiotics might actually be easier than ensuring the safety of live bacteria [49]. In addition, postbiotics do not transfer any resistance genes [50]. These features consolidate *E. faecalis* EF-2001′s position as a potentially safer choice in the probiotic market, where consumers may have underlying health conditions or sensitivities [51].

## 4. Materials and Methods

### 4.1. Materials and Chemicals

*E. faecalis* EF-2001L was originally obtained from Nihon BRM Co. Ltd. (Tokyo, Japan). Antibiotic strips were purchased from Liofilchem (Roseto degli Abruzzi, Italy). Amino acids such as L-arginine, L-histidine, L-lysine, L-ornithine, L-tryptophan, and L-tyrosine were purchased from Sigma-Aldrich (Seoul, Republic of Korea). Pancreatin was ordered from Daejung Chemicals (Siheung, Republic of Korea). Pepsin was purchased from Roche (Seoul, Republic of Korea). All media used for cultivation were obtained from Becton Dickinson (Sparks, NV, USA) unless specified.

### 4.2. Gene Stability Test

In this study, *E. faecalis* EF-2001L was initially cultivated by selecting a fresh single colony and inoculating it into MRS broth, followed by incubation at 37 °C for approximately 18 h to reach a concentration of approximately 10^9^ CFU/mL, constituting the 1st generation. To obtain subsequent generations, 1% of the inoculum from the 1st generation culture was transferred into fresh MRS broth and incubated under the same conditions, forming the 2nd generation. This subculturing process was repeated every 8 to 10 h, with 1% of the inoculum from each preceding generation used to inoculate fresh broth until the 25th generation was achieved [52]. Finally, both the 1st and 25th generations of *E. faecalis* EF-2001L cultures were used for gDNA isolation.

Then, gDNA was extracted for long and short read whole genome sequencing (WGS) using Qiagen’s MagListoTM 5M Genomic DNA Extraction Kit. PacBio Sequel System (Pacific Biosciences, USA) and Illumina platform (Illumina, San Diego, CA, USA) were used for the WGS [53]. Using a DNA 1000 chip, an Agilent 2100 Bioanalyzer was used to confirm the quality and amount of the DNA. The SMRTbell Express kit v2.0 (PacBio) was utilized to generate a library after 3 μg of gDNA was sequenced using single-molecule real-time (SMRT) technology [54]. De novo assembly was performed using the Microbial Assembly application in SMRT Link v10.2 (PacBio). Following de novo assembly, PacBio sequence errors were corrected using Illumina sequencing, and using the TruSeq Nano DNA library preparation kit (Illumina, USA), more accurate contigs were developed. After assembly, Trimmomatic v0.38 was used with default settings to perform error correction and adapter trimming on the Illumina short-read reads [55]. Then, paired-end data were corrected using Pilon v1.21 and mapped against the PacBio assembly using BWA-MEM v0.7.17 [56]. The assembly raw reads were mapped and annotated based on the Prokka (version 1.12b) and EggNOG (version 4.5) databases to estimate the gene coding information [57,58]. Further mobile genetic elements and their relationship to antimicrobial resistance genes and virulence factors were found at MobileElementFinder [59]. IslandViewer 4 was used to locate the gene of interest and MGEs in the *E. faecalis* EF-2001L genome [60].

### 4.3. Biosynthetic Gene Predictions

The whole genome sequence data of *E. faecalis* EF-2001L was obtained from our research collaborators [7]. Genes related to biosynthetic pathways, essential amino acids, vitamins, and stress-related proteins were identified in the *E. faecalis* EF-2001L genome using the Rapid Annotratins using Subsythemes Technology (RAST) server [20]. The Carbohydrate-Active enZYmes (CASy) database was used to identify the carbohydrate metabolism ability of the *E. faecalis* EF-2001L strain [61].

### 4.4. In Vitro Safety Assessment of E. faecalis EF-2001L

#### 4.4.1. Antibiotic Susceptibility of *E. faecalis* EF-2001L

The antibiotic susceptibility of *E. faecalis* EF-2001L was determined with previously described methods with slight modifications by using MIC test strips (Liofilchem, Roseto degli Abruzzi, Italy) of nine antibiotics, i.e., ampicillin, chloramphenicol, clindamycin, erythromycin, gentamicin, kanamycin, streptomycin, tetracycline, and vancomycin [27]. *E. faecalis* EF-2001L was streaked on MRS agar and incubated at 37 °C for 72 h. The single colony of *E. faecalis* EF-2001L was then inoculated into MRS broth at 37 °C for 24 h. Overnight culture was fully spread on MRS agar. MIC test strip was fixed on the surface of the agar plate. Inhibition zones produced by nine antibiotic strips were measured after 24 h incubation. The European Food Safety Authority’s (EFSA) recommendations were followed in order to evaluate the antibiotic susceptibility [62].

#### 4.4.2. Hemolytic Activity

Hemolytic activity on *E. faecalis* EF-2001L was tested according to the method specified by Rastogi et al. [63]. Overnight grown bacterial culture was streaked onto a 5% sheep blood agar plate (MB cell, Seoul, Republic of Korea), and incubated at 37 °C for 48 h. α-, β-, or γ-hemolysis were noted based on the clearing zone’s appearance around the colonies. As a positive control, *S. aureus* ATCC 6538 was employed.

#### 4.4.3. Biogenic Amine Production Assay

Biogenic amine production from *E. faecalis* EF-2001L was observed using the method described previously [10]. *E. faecalis* EF-2001L cultured in MRS broth overnight was inoculated on MRS agar supplemented with 0.1% of six precursor amino acids (L-arginine, L-histidine, L-lysine, L-ornithine, L-tryptophan, and L-tyrosine). After 96 h incubation under aerobic conditions, color change in the solid media was monitored. The exhibition of purple color in the medium leads to the determination of the biogenic amine production.

#### 4.4.4. Bile Salt Deconjugation

Bile salt deconjugation was examined on *E. faecalis* EF-2001L by the method described in Dashkevicz et al. with slight modification [64]. *E. faecalis* EF-2001L was streaked on MRS agar, including 0.5% sodium taurodeoxycholate (TDCA-MRS agar). After 24 h incubation, plates were monitored for clear zones around the colonies. The presence of a clear zone indicates the bile salt hydrolysis (BSH) activity from *E. faecalis* EF-2001L.

#### 4.4.5. Acid Tolerance Test

The acid tolerance test of EF-2001L was performed using a previously described method [65]. Overnight *E. faecalis* EF-2001L culture in MRS broth (10^8^ CFU/mL) was centrifuged (4000 RPM, 5 min). After washing twice with 1 × PBS (pH 7.4), the pellet was resuspended in MRS medium, where pH was adjusted to 2, 3, or 4 with 1N HCl and 1N NaOH solutions. To assess the stability of *E. faecalis* EF-2001L cells at different pH levels, 100 μL of bacterial suspension was incubated at 37 °C for 24 h. Following this, the suspension was spread onto MRS agar plates and incubated for an additional 24 h at 37 °C under aerobic conditions. CFU/mL were then determined using the colony plate counting method.

#### 4.4.6. Simulated Gastrointestinal Condition Tolerance Test

Survival of *E. faecalis* EF-2001L was tested in the simulated gastric fluid (SGF) as well as the simulated intestinal fluid (SIF) according to Charteris et al. with slight modification [66]. Overnight culture of *E. faecalis* EF-2001L (10^8^ CFU/mL) in MRS broth at 37 °C for 24 h was centrifuged (4000 RPM, 8 min), and the pellet was resuspended in either SIF (1 g/L pancreatin in 1 × PBS, pH 7 and 8) or SGF (3 g/L pepsin in 1 × PBS, pH 2, 3, and 4). The pellet was rinsed twice with 1 × PBS (pH 7.4). Five cell suspensions were incubated for 3 h, and 100 μL of them were individually spread on MRS agar. After 24 h incubation, the viable cell rate was calculated using the following equation:

$$Viable\;cell\;rate\left(\%\right)=\frac{{Log}_{10}\;value\;of\;survived\;cells}{{Log}_{10}\;value\;of\;initial\;cells}\times 100$$

#### 4.4.7. Gelatin Hydrolysis Assay

The gelatin hydrolysis assay was performed by adopting the method from Pickett et al. with a slight modification [67]. *E. faecalis* EF-2001L was inoculated into MRS broth overnight. Then, 1% of *E. faecalis* EF-2001L culture was inoculated into gelatin nutrient medium (MRS with 12 g/L gelatin, 5 g/L soy peptone, and 30 g/L beef extract). Gelatin nutrient medium without inoculum was observed as a negative control. The test gelatin nutrient medium as well as the negative control were then incubated at 37 °C for eight days. The cultures were cooled on the ice for 15 min, and solidification was determined at the slant position.

#### 4.4.8. Hyaluronidase Assay

The hyaluronidase assay was examined using the method described by Hynes et al. [68]. Modified Brain-Heart Infusion Hyaluronic Acid (BHI-HA) medium was prepared with BHI medium including 1% agar as well as 400 μg/mL sodium hyaluronate and 1% bovine albumin fraction V. *E. faecalis* EF-2001L was inoculated into MRS broth. After 24 h incubation, 20 μL of bacterial inoculum was dropped onto the surface of modified BHI-HA agar and incubated at for 24 h. 2N acetic acid was poured into the plate for 10 min of staining, and hyaluronidase activity was determined with the presence of a clear zone around the bacterial colony. *S. aureus* ATCC 6538 was used as a positive control.

#### 4.4.9. D-lactate Production Test

The D-lactate production test was performed on *E. faecalis* EF-2001L following the method described by Hu et al. The D-lactate levels were measured using a colorimetric D-lactate assay kit (Abcam, Cambridge, UK), following the manufacturer’s instructions [69]. *E. faecalis* EF-2001L from overnight culture was harvested and washed with cold 1 × PBS. The pellet was resuspended in assay buffer and then homogenized. After centrifugation (10,000× *g*, 10 min), the supernatant was collected and prepared with assay buffer in a 96-well plate. The reaction mix was added on the sample and then incubated for 30 min at room temperature in the dark area. Under measuring absorbance at 450 nm, the concentration of the sample was calculated based on a standard curve, which was prepared with D-lactate standard solution.

Carbohydrate fermentation of the *E. faecalis* EF-2001L was evaluated using the API 50 CHL kit (Bio-merieux, Lyon, France) as per the manufacturer’s instructions. The results were interpreted by the color change referred to by the color chart provided by the manufacturer.

#### 4.4.10. Aggregation

Auto-aggregation and co-aggregation were tested on *E. faecalis* EF-2001L using the method referred to by Solieri et al. [70]. The overnight EF-2001L culture was centrifuged (4000 RPM, 5 min) and washed twice with sterile water. Both *E. faecalis* EF-2001L and EF-2001HK cells, resuspended in 1 × PBS with OD_600_ as 1.0, were vortexed for 10 s and then incubated at RT for 5 h. For the Heat-Killed (HK) cells, the postbiotic product EF-2001^®^, which is derived from human feces, was obtained as a commercially purified parabiotic product from Bereum Co., Ltd. (Wonju, Republic of Korea). This product was provided as an HK dried powder, and the dried EF-2001HK contained 7.5 × 10^12^ cells per gram [48]. 100 μL from the surface of each suspension was mixed with 500 μL PBS, and the absorbance was measured. The auto-aggregation rate was calculated by the following equation:

$$Auto\text{-}aggregation\;rate\left(\%\right)=\frac{{Abs}_{0h}-{Abs}_{5h}}{{Abs}_{0h}}\times 100$$

where ${Abs}_{0h}$ refers to initial absorbance and ${Abs}_{5h}$ refers to absorbance after 5 h of incubation of each *E. faecalis* EF-2001L or EF-2001HK suspensions.

Pathogenic strains such as *S. aureus* ATCC 6538 and *E. coli* LF82 were used as indicator bacteria for co-aggregation tests. Suspensions were prepared as previously described. *E. faecalis* EF-2001L was mixed with each pathogenic indicator suspension in equal volumes and vortexed for 10 s. Each single bacterial suspension was used as a control. After 5 h incubation at RT, the absorbances of single and mixture suspensions were measured. By following the equation, co-aggregation was calculated:

$$Co\text{-}aggregation\;rate\left(\%\right)=1-\frac{{Abs}_{\left[EF+PA\right]}}{{[Abs}_{EF}-{Abs}_{PA}]/2}\times 100$$

where ${Abs}_{EF}$ refers to the absorbance of EF-2001L or EF-2001HK, ${Abs}_{PA}$ refers to the absorbance of a single pathogen, and ${Abs}_{\left[EF+PA\right]}$ refers to the absorbance of a mixture of EF-2001L or EF-2001HK and a pathogen.

#### 4.4.11. Morphological Visualization by Scanning Electron Microscopy

To evaluate cell morphology, we employed scanning electron microscopy (SEM). A single fresh colony was first put into MRS medium and allowed to incubate for a full day at 37 °C. Then, 300 μL of bacterial suspension at a cell density of 10^6^ cells/mL was added to each well in a 96-well plate, and silicon wafers, each measuring around 0.5 × 0.5 cm, were inserted into each well. These plates were incubated without any shaking overnight. The bacterial cells were fixed with 5% glutaraldehyde at 4 °C for an overnight period. Following that, there was a six-hour post-fixation with osmium tetroxide. After washing, the samples were prepared for SEM visualization using the specified methods [71]. To prepare the sample, 0.1 g of *E. faecalis* EF-2001HK was diluted 10^2^ times by adding it to 10 mL of 1 × PBS buffer. The mixture was then vortexed for at least 1 min and subsequently sonicated for 1 h in an ultrasonic cleaner. Finally, the suspension was further diluted 10^6^ times for SEM analysis.

#### 4.4.12. Cytotoxicity

The cytotoxicity of *E. faecalis* EF-2001HK was evaluated using the Quanti-LDH™ PLUS Cytotoxicity Assay Kit (BIOMAX, Guri, Republic of Korea) with slight modifications to the reference method [72]. Human colorectal adenocarcinoma HT-29 cells were purchased from AddexBio Technologies, San Diego, CA, USA. The cells (1 × 10^5^ cells/well) were cultured in Dulbecco’s Modified Eagle’s Medium (DMEM) supplemented with 10% fetal bovine serum (FBS) and 100 U/mL penicillin–streptomycin solution. The cells were incubated at 37 °C and 5% CO_2_ for 24 h. After this incubation, the medium was removed, and the cells were washed twice with PBS containing 1% (*w/v*) bovine serum albumin (BSA). Fresh medium was then added to each well.

*E. faecalis* EF-2001HK was prepared at concentrations ranging from 1 × 10^7^ to 1 × 10^9^ cells/well and added to the HT-29 cells. The cells were incubated with *E. faecalis* EF-2001HK for an additional 24 h at 37 °C and 5% CO_2_. Following incubation, 100 μL of LDH reaction mix was added to each well, and the plates were incubated at room temperature for 30 min in the dark. A lysate solution was used as a positive control, and a vehicle treatment group served as the negative control. After adding 10 μL of stop solution to each well, the absorbance was measured at 490 nm using a colorimetric microplate reader to determine cytotoxicity.

#### 4.4.13. pH Stability Assay of *E. faecalis* EF-2001HK

For the pH stability tests, the dried powder of *Enterococcus faecalis* EF-2001HK was utilized. Eight different pH buffer solutions were prepared, including hydrochloric acid buffer (pH 2.0), acetate buffers (pH 3.0 to 6.0), phosphate buffer (pH 7.0), and alkaline borate buffers (pH 8.0 and 9.0). To each buffer, 20 μg/mL of tetracycline and 10 μg/mL of cycloheximide antibiotics were added to prevent microbial contamination. Subsequently, 0.1 g of the *Enterococcus faecalis* EF-2001HK was mixed with 10 mL of each buffer solution. The resulting suspensions were subjected to vortexing for 1 min, sonication for 1 h in an ultrasonic water bath, and then incubated at room temperature for either 7 or 15 days. At the end of the incubation periods, the cell suspensions were serially diluted up to 10^4^ in 1 × PBS. A 4 μL aliquot of the diluted cell suspension was then pipetted into the chambers of a bacterial hematocytometer (Erma, Tokyo, Japan), and cell counts were performed at 400× magnification using a microscope (Leica, Wetzlar, Germany) on 0, 7, and 15 days. The hematocytometer’s counting chamber is divided into eight large sections, with each large section containing 16 small sections. The total cell count was calculated using the following formula:

Cell number = (Ct × 16 (No. of small sections))/(8 (No. of large sections)) × 10^6^ (dilution factor) × 50 (room depth) × 10^3^ (room volume)

where Ct represents the total number of bacterial cells counted in the eight large sections.

### 4.5. Statistical Analysis

All experiments were conducted thrice or more, and the results are presented as the mean ± standard deviation. The Student’s *t*-test was used to determine the difference between the treated and untreated samples. Significant changes are denoted in the figures with asterisks when *p* < 0.05.

## 5. Conclusions

*E. faecalis* EF-2001L demonstrates considerable promise as a probiotic, with genomic characteristics that enhance gut health without producing harmful biogenic amines. Its safety profile, highlighted by minimal virulence, low D-lactate production, and general antibiotic susceptibility, makes it a strong candidate for therapeutic applications, though careful monitoring of its resistance to certain aminoglycosides is necessary. The strain’s resistance in simulated gastrointestinal environments, along with its lack of bile salt deconjugation and hemolytic activity, further supports its potential as a probiotic. Given its lack of horizontal gene transfer and the absence of gene transfer due to its postbiotic nature, the postbiotic *E. faecalis* EF-2001HK could be a better choice for use in food and dietary supplements. Future research could focus on understanding EF-2001HK interactions within the human microbiome, its long-term impact on metabolic and overall health, and its potential role in preventing chronic diseases. As the field of microbiome research advances, *E. faecalis* EF-2001L has the potential to become a significant player in the next generation of probiotic and postbiotic therapies.

## Figures and Tables

**Table 1:** Genetic characteristics of the whole genome sequence of the 1st and 25th generations of live *E. faecalis* EF-2001.

**Taxon Name**	***Enterococcus faecalis*** **EF-2001**	
**Strain ID**	**1^st^ Generation**	**25^th^ Generation**
Status	Complete	Complete
Genome Size (bp)	2,806,628	2,806,355
GC content (%)	37.7	37.6
No. of contigs	1	1
No. of CDSs	2544	2528
No. of RNA genes	76	76
Homology of EF-2001 1st and 25th generation by OrthoANI analysis	99.97%	

**Table 2:** The list of CAZyme genes matched in the EF-2001 genome.

CAZy Families	Number of Genes	Sub-Category
Auxiliary activities (AAs)	2	AA10(2)
Carbohydrate-binding modules (CBMs)	5	CBM34(2), CBM50(3)
Carbohydrate esterase (CEs)	5	CE1(1), CE4(1), CE7(1), CE9(2)
Glycoside hydrolases (GHs)	49	GH1(8), GH109(1), GH125(1), GH126(1), GH13(3), GH154(2), GH170(3), GH177(2), GH179(2), GH18(2), GH2(1), GH20(1), GH24(1), GH25(1), GH3(1), GH32(1), GH35(1), GH38(1), GH4(1), GH130(2), GH63(1), GH65(2), GH73(5), GH88(2), GH92(1), GH94(1), GH136(1)
Glycosyltransferases (GTs)	19	GT2(12), GT26(1), GT27(1), GT28(1), GT4(1), GT51(3)
Polysaccharide lyases (PLs)	ND	ND

**Table 3:** Associated biosynthetic genes detected in the EF-2001L genome.

Category	Sub-Category	Protein	GO
Essential Amino acids	Tryptophan	Tryptophan synthase alpha chain TrpEa, Tryptophan synthase beta chain TrpEb	GO:0004834
	Methionine	Methionine transporter MetT, MetN, MetP; Methionine ABC transporter ATP-binding protein	GO:0006814, GO:0006885, GO:0015385, GO:0016021 GO:0005215, GO:0006810, GO:0016020 GO:0005524, GO:0016887
	Arginine	Arginine decarboxylase, SpeA	GO:0008792, GO:0008792
	Threonine	Threonine synthase, ThrC	GO:0004795
Vitamins	Thiamine	Thiamin ABC transporter, ATPase component Homocysteine S-methyltransferase	GO:0005215, GO:0006810 GO:0016020 GO:0005524, GO:0016887 GO:0008898 GO:0006814, GO:0006885, GO:0015385, GO:0016021
	Biotin	Biotin synthase, BioB	GO:0004076
	Folate	Dihydrofolate synthase FolCDHFS, Dihydrofolate reductase Dhfr0	GO:0008841 GO:0004146

GO: Gene Ontology.

**Table 4:** Associated stress-responsive genes in the EF-2001 genome.

Category	Protein	GO
Oxidative stress response	Glutathione peroxidase GPX Glutathione reductase GR Glutathione synthetase gshB	GO:0004602 GO:0004362 GO:0004363
Heat stress response	Heat shock protein GrpE Chaperone protein DnaJ	GO:0000774, GO:0006457, GO:0042803, GO:0051087 GO:0006457, GO:0031072, GO:0051082
Osmotic stress	Aquaporin Z	GO:0005215, GO:0006810, GO:0016020

**Table 5:** Minimal inhibitory concentration (MIC) and antibiotic susceptibility of EF-2001.

Antibiotic	MIC (µg/mL)	Cut-off Value (µg/mL)	Assessment
Ampicillin	0.25	2	S
Vancomycin	1	2	S
Gentamycin	24	32	S
Kanamycin	1024	1024	R
Streptomycin	1024	128	R
Erythromycin	1.5	4	S
Clindamycin	1	4	S
Tylosin	4	4	S
Tetracycline	0.1	4	S
Chloramphenicol	4	16	S

S, susceptible; R, resistant.

## Data Availability

Data is contained within the article and Supplementary Material.

## Supplementary Materials

The following supporting information can be downloaded at: https://www.mdpi.com/article/10.3390/ph17101383/s1, Figure S1: Phylogenetic trees were constructed for EF-2001 reference (EF-2001), 1st generation and 25th generation genomes; Figure S2: The positions of the resistance gene and the nearest MGEs; Table S1: Biogenic amine production by EF-2001; Table S2: Hyaluronidase activity of EF-2001; Table S3: List of Mobile Genetic Elements hits in *E. faecalis* EF-2001 genome.
