# Supplementary material for: Probiotic and Postbiotic Potentials of Enterococcus faecalis EF-2001: A Safety Assessment
Source: Pharmaceuticals (Basel). 2024 Oct 17;17(10):1383. doi: 10.3390/ph17101383 (PMC11510163; doi:10.3390/ph17101383)
Supplement: Supplementary file 1 [file pharmaceuticals-17-01383-s001.zip › pharmaceuticals-3206679-supplementary.pdf]

pharmaceuticals

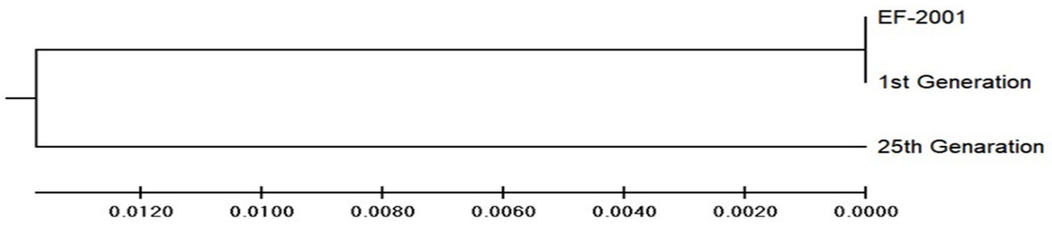

Figure S1. Phylogenetic trees were constructed for EF-2001 reference (EF-2001), 1st generation and 25th generation genomes.

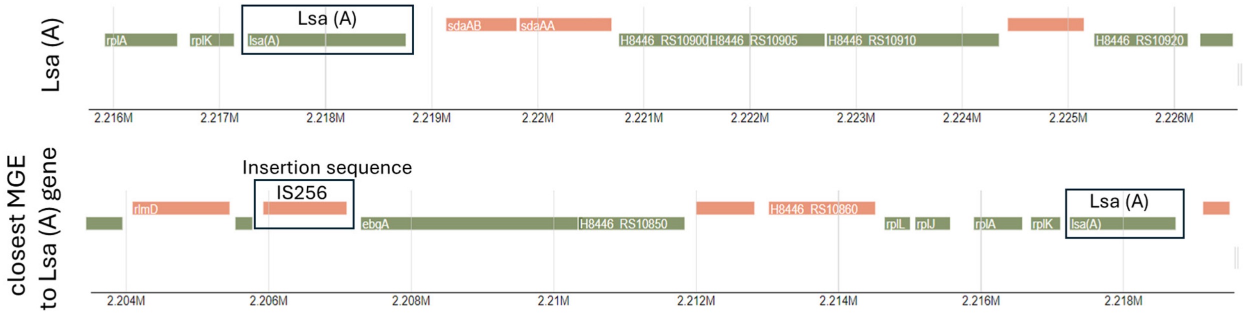

Figure S2. The positions of the resistance gene and the nearest MGEs .

Table S1. Biogenic amine production by EF-2001.

No	Amines	Results
1.	L-arginine	Negative
2.	L-histidine	Negative
3.	L-lysine	Negative
4.	L-ornithine	Negative
5.	L-tryptophan	Negative
6.	L-tyrosine	Negative

Table S2. Hyaluronidase activity of EF-2001.

Strain	Hyaluronidase activity
E. faecalis EF-2001	(-)
S. aureus ATCC 6538	(+)

Clear zones around colonies indicate positive hyaluronidase activity.

Table S3. List of Mobile Genetic Elements hits in *E. faecalis* EF-2001 genome.

MGE No	Name	Prediction method	Type	Allele length	Identity
1	ISEfm2	Alignment to reference	Insertion sequence	1325	0.9969811
2	ISEfm2	Alignment to reference	Insertion sequence	1325	0.9977358
3	ISEfm2	Alignment to reference	Insertion sequence	1325	0.9977358
4	ISEfm2	Alignment to reference	Insertion sequence	1325	0.9977358
5	ISEfm2	Alignment to reference	Insertion sequence	1325	0.9977358
6	ISEfm2	Alignment to reference	Insertion sequence	1325	0.9977358
7	ISEfm2	Alignment to reference	Insertion sequence	1325	0.9977358
8	ISEfm2	Alignment to reference	Insertion sequence	1325	0.9977358
9	ISEfm2	Alignment to reference	Insertion sequence	1325	0.9977358

10	IS1062	Alignment to reference	Insertion sequence	1062	0.9981168
11	IS1062	Alignment to reference	Insertion sequence	1062	0.9981168
12	IS1062	Alignment to reference	Insertion sequence	1062	0.9981168
13	IS1062	Alignment to reference	Insertion sequence	1062	0.9990584
14	IS1062	Alignment to reference	Insertion sequence	1038	0.9980732
15	cn_24452_ISEfm2	inferred	Composite transposon	24,452	0.9977358
16	cn_9853_IS1062	inferred	Composite transposon	9853	0.9980732
17	cn_35042_IS1062	inferred	Composite transposon	35,042	0.9981168
